# Clinical Significance of Somatostatin Receptor (SSTR) 2 in Meningioma

**DOI:** 10.3389/fonc.2020.01633

**Published:** 2020-09-03

**Authors:** Wei Wu, Yunxiang Zhou, Yali Wang, Lihong Liu, Jianyao Lou, Yongchuan Deng, Peng Zhao, Anwen Shao

**Affiliations:** ^1^Department of Medical Oncology, First Affiliated Hospital, School of Medicine, Zhejiang University, Hangzhou, China; ^2^Department of Surgical Oncology, The Second Affiliated Hospital, School of Medicine, Zhejiang University, Hangzhou, China; ^3^Department of Radiation Oncology, The Second Affiliated Hospital, School of Medicine, Zhejiang University, Hangzhou, China; ^4^Department of General Surgery, The Second Affiliated Hospital, School of Medicine, Zhejiang University, Hangzhou, China; ^5^Department of Neurosurgery, The Second Affiliated Hospital, School of Medicine, Zhejiang University, Hangzhou, China

**Keywords:** meningioma, SSTR2, somatostatin, somatostatin analogs, diagnosis, treatment, prognosis

## Abstract

Somatostatin receptor (SSTR) 2, widely expressed in meningioma, is a G-protein-coupled receptor and can be activated by somatostatin or its synthetic analogs. SSTR2 is therefore extensively studied as a marker and target for the diagnosis and treatment of meningioma. Accumulating studies have revealed the crucial clinical significance of SSTR2 in meningioma. Summarizing the progress of these studies is urgently needed as it may not only provide novel and better management for patients with meningioma but also indicate the direction of future research. Pertinent literature is reviewed to summarize the recent collective knowledge and understanding of SSTR2’s clinical significance in meningioma in this review. SSTR2 offers novel ideas and approaches in the diagnosis, treatment, and prognostic prediction for meningioma, but more and further studies are required.

## Introduction

Meningiomas, arising from the dura mater of the brain and spinal cord, are currently the most frequent primary intracranial tumors (1). Meningiomas have an estimated annual incidence of 7.86 cases per 100,000 people, accounting for up to 30% of all primary intracranial tumors (2–4). The majority of meningiomas are histologically benign and slow growing and correspond to World Health Organization (WHO) grade I, while up to 20% of the tumors are classified as WHO grade II or grade III meningiomas on account of features of increased malignancy and local invasiveness (5, 6). Progressive enlargement of the tumor and compression of adjacent neural tissue lead to clinical manifestations, such as generalized or focal seizure disorders, focal neurological deficits, and neuropsychological decline (3). The preliminary radiological diagnosis and precise localization of meningioma mainly depend on magnetic resonance imaging (MRI) nowadays (1, 7). Surgical resection remains the standard treatment for meningiomas; however, observation should be considered as a therapeutic option if the clinical situation permits; meanwhile, radiotherapy is becoming increasingly important in the treatment of meningiomas, especially for those surgically inaccessible tumors; in addition, large-scale clinical trials for pharmacotherapy have not presented positive results yet (1, 8–10).

Somatostatin receptors 1–5 (SSTR1–5) pertain to the family of seven-transmembrane G protein-coupled receptors and are widely expressed in both normal tissues and solid tumors (11, 12). These five receptors share some common features underlying structure and signaling mechanisms, but their cellular/subcellular localization and mode of regulation vary from one to another (12, 13). Among these receptors, the overexpression of SSTR2 was the most frequent in meningiomas compared with the other SSTR subtypes (14). In recent years, accumulating studies have reported the correlation between SSTR2 expression and meningiomas. However, to the best of our knowledge, no literature review has been published to summarize it thus far. Hence, we provide a detailed summary of the current understanding of the clinical significance of SSTR2 in meningioma in this review.

## Synopsis of SSTR2

The encoding gene for SSTR2 is localized at chromosome 17q25.1 and comprises two exons. The first exon contains the 5′ untranslated region while exon 2 contains the entire coding region and 3’ untranslated region (13). The SSTR2 gene has a strong tolerance to sequence variations; hardly any disease-related mutations have been discovered in the SSTR2 gene (13, 15). The transcribed SSTR2 mRNA is spliced to produce two isoforms of SSTR2 named SSTR2A (the long form) and SSTR2B (the short form), which differ in the length of the cytoplasmic tail (12, 16). Human tissues include the SSTR2A variant exclusively (13). Typical seven-transmembrane segments and four putative N-glycosylation sites could be displayed in the SSTR2 protein of 369 amino acids (13). The protein can be detected by Western blot as a characteristic band of 70–80 kDa (13, 17, 18). SSTR2 is ubiquitously distributed in normal tissues especially in the central nervous system (CNS) and endocrine system (12, 19–21). SSTR2 is also expressed widely and represents manifold functions in various tumor tissues including neuroendocrine tumors, pituitary adenomas, breast cancer, melanoma, thyroid cancer, and meningioma (20, 22–25). Nevertheless, the expression level of SSTR2 between normal tissues and tumor tissues is different. For instance, SSTR2 was identified as significantly highly expressed in meningioma tissues compared with normal tissues by Anne et al. (26). The expression of SSTR2 can routinely be detected through reverse-transcription polymerase chain reaction and immunohistochemistry (Table 1); the vast majority of meningiomas express SSTR2 (14, 25–32). SSTR2 mediates diverse physiological effects when activated by somatostatin or its synthetic analogs, such as regulating the physiologic secretion of insulin, glucagon, thyroid-stimulating hormone, and growth hormone (GH); protecting retina nerves; and regulating neuronal excitability (13, 23, 33–36).

**TABLE 1:** Studies regarding the detection methods and expression of SSTR2 in meningiomas.

Subjects										
No. of meningiomas	40	60	20	50	42	22	26	35	68	148
Detection methods	IHC	IHC	RT-PCR	RT-PCR	RT-PCR	IHC	RT-PCR	IHC	IHC	IHC
SSTR2 expression (%)*	70	100	100	100	79	64	100	74	87	100
References	(14)	(25)	(26)	(27)	(28)	(29)		(30)	(31)	(32)

*The results stand for the percentage of SSTR2-expressing meningiomas. IHC, immunohistochemistry; RT-PCR, reverse transcription-polymerase chain reaction.

## SSTR2-Related Diagnosis Approaches for Meningioma

**FIGURE 1:**
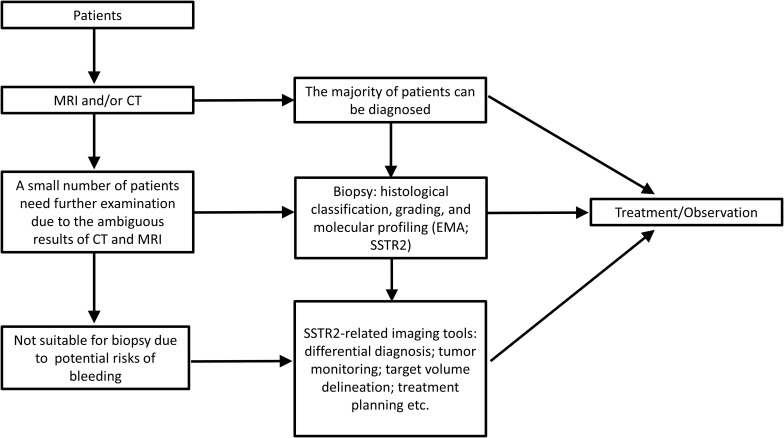
The diagnosis process and application scenarios of meningioma with the utilization of SSTR2.

A preliminary diagnosis of meningioma typically relies on MRI and computed tomography (CT); further diagnosis includes histological classification, grading, and molecular features (1, 3, 7). However, because the results of CT and MRI are sometimes ambiguous and because biopsy carries potential risks of bleeding, additional approaches (Figure 1) for the diagnosis of meningioma are needed (37). Besides, this “integrated diagnosis” era also calls for other novel and efficient diagnostic methods for accurate diagnoses of meningioma (38, 39).

Somatostatin receptor 2A was found to be a more sensitive diagnostic marker for meningioma than epithelial membrane antigen—a conventional meningioma marker (40). Since then, accumulating evidence has emerged to support the diagnosis value of SSTR2A as it is a highly sensitive and specific marker for meningioma (41, 42). A case report has shown that SSTR2A, combined with epithelial membrane antigen, provides assistance for the diagnosis of an unusual skull tumor with psammomatoid bodies (43).

Moreover, given that SSTR2 is expressed in almost 100% meningiomas (14, 26, 30, 44, 45), radiolabeled SSTR2 ligands have been widely utilized in the modern radiological diagnosis of meningioma (Table 2).

**TABLE 2:** Clinical studies using radiolabeled SSTR2 ligands for meningioma diagnosis.

Image diagnostic method	Radiolabeled SSTR2 ligand	Sample size	Major/novel functions	References
PET	^68^Ga-DOTATATE	21 pts (81 Ms)	Discriminating meningioma and tumor-free tissue even in recurrent tumors after previous therapy.	(46)
		64 Ms	Selecting the time point for treatment initiation; predicting tumor growth rate	(48)
		30 pts (49 Ms)	Discriminating meningioma and post-treatment change; improving diagnosis and extent of disease evaluation.	(50)
	^68^Ga-DOTATOC	3 pts (8 Ms)	Offering excellent imaging properties and a very high tumor-to-background ratio even in small meningiomas.	(49)
		21 pts	Showing higher specificity for meningioma diagnosis than FET PET.	(54)
PET/CT	^68^Ga-DOTATATE	82 pts	Improving detection of the transosseous extension of intracranial meningiomas.	(53)
	^68^Ga-DOTATOC	26 pts	Improving target volume delineation for IMRT especially for skull base meningioma and recurrent disease after surgery	(47)
		134 pts	Providing additional information in patients with uncertain or equivocal results on MRI; helping to confirm MRI-based diagnosis of meningiomas in cases of biopsy limitations.	(52)
PET/MRI	^68^Ga-DOTATOC	10 pts	Sketching treatment target volume; benefiting radiosurgical treatment planning.	(51)
SPECT SRS	^111^In-octreotide	27 pts	Discriminating meningioma and nonspecific hyperperfusion; displaying remaining tumor tissue or relapse of meningioma in postsurgical follow-up.	(60)
		22 pts	Detecting Ms with an extremely high sensitivity (100%).	(62)
		47 pts	Discriminating Ms and other CNS tumors, combined with MRI.	(63)
		70 pts	Discriminating Ms and other tumors, postoperative scar or radionecrosis at the skull base.	(64)
		95 pts	Discriminating Ms and other CNS tumors.	(65)
		50 pts	Discriminating Ms and other cranial dural-based lesions, combined with MRI.	(66)
SPECT/CT SRS	^99m^Tc-HYNIC-octreotide	30 pts	Showing high meningioma radioactivity accumulation with a sensitivity of 100 %.	(70)

*CNS, central nervous system; DOTATATE, DOTA-D-Phe1-Tyr3-octreotate; DOTATOC, DOTA-(Tyr3)-octreotide; FET, fluoro-ethyl-tyrosine; IMRT, intensity modulated radiotherapy; ^111^In, 111-Indium; MRI, Magnetic Resonance Imaging; Ms, meningiomas; PET, Positron emission tomography; pts, patients; SPECT, single-photon emission computed tomography; and SRS, Somatostatin receptor scintigraphy.*

Positron emission tomography (PET)-based imaging (including PET, PET/CT, and PET/MRI) applying radiolabeled somatostatin agonists such as ^68^Ga-DOTATATE (DOTA-D-Phe1-Tyr3-octreotate) and ^68^Ga-DOTATOC (DOTA-[Tyr3]- octreotide) has been presented to be a precise diagnostic means; this technology is helpful in target volume delineation, radio/surgical treatment planning, diagnosing small meningiomas, and monitoring tumor growth rate, etc. (46–51). Recent researches demonstrated a higher sensitivity of ^68^Ga-DOTATOC or ^68^Ga-DOTATATE PET or PET/CT by comparison with contrast-enhanced MRI or fluoroethyl-tyrosine PET in diagnosing meningiomas (52–54). Additionally, the exact delineation seems challenging in some cases with low CT and MRI contrast as a result of osseous infiltration or in skull base meningiomas. PET-based imaging with radiolabeled SSTR2 ligands shows superiority in overcoming this diagnostic difficulty due to the highly specific binding of SSTR2 ligands to SSTR2 in meningiomas and the extremely low absorption in adjacent structures such as bone and brain tissue (7, 53, 55, 56). Furthermore, in the case of atypical meningioma or a rare type of meningioma like optic nerve sheath meningioma, SSTR2-related PET/CT is also deemed to be a useful noninvasive diagnostic method (57–59).

Single photon emission CT (SPECT) somatostatin receptor scintigraphy (SRS) using ^111^In-octreotide is another valuable tool for the diagnoses of meningiomas based on the general expression of SSTR2 in all meningiomas. SPECT SRS with ^111^In-octreotide is considered a highly specific imaging approach, and it plays an important role in post-treatment follow-up in meningioma patients (60, 61). Hildebrandt et al. have shown that *in vivo* detection of SSTRs by ^111^In-octreotide scintigraphy in meningioma patients had a high sensitivity as a high density of SSTRs was detected in all cases (62). Regarding differential diagnosis in meningioma and other CNS tumors such as craniopharyngiomas, schwannomas, and ependymomas or other cranial dural-based lesions, SPECT SRS with ^111^In-octreotide has also proven its values (63–66). In the meantime, SPECT SRS could offer aid in the differential diagnosis between meningiomas and radionecrosis or postoperative scar at the skull base, which is meaningful for recurrence screening of meningioma (64). As for cases with an atypical presentation, SPECT SRS can offer support in distinguishing optic nerve sheath meningioma from alternative orbital masses (67, 68).

Other SSTR2-related imaging tools also exhibit diagnostic values. For instance, SPECT/CT SRS using ^99m^Tc-HYNIC-octreotide specifically binding to SSTR2 in meningioma can diagnose primary optic nerve sheath meningioma or allow differentiation of meningiomas from inactive pituitary adenomas, which is seemingly elusive by conventional MRI (69, 70).

These studies suggest that SSTR2-related imaging tools with radiolabeled somatostatin agonists are valuable for precise-positioning tumor detection, evaluation of disease extension, differential diagnosis, and tumor monitoring even in small, asymptomatic, or rare cases.

## SSTR2-Related Treatment Approaches for Meningioma

Individualized precision treatment regimens should be employed in treating patients with meningioma since heterogeneity between meningiomas exists and clinical outcomes for different patients vary greatly (1). Correct decision making in the management of meningioma patients is significant in order to achieve optimal clinical consequence and long-time survival (71–73). Surgery is the main treatment for most meningiomas; however, effective treatment modalities for patients with unresectable or recurrent meningioma remain elusive. It is of interest that SSTR2-related/targeted treatments could provide novel therapeutic interventions against meningiomas beyond traditional therapies, especially for those inoperable or recurrent patients.

The exact biological function of SSTR2 in meningioma is hitherto not very sharply defined, but its activation may be correlated to an antiproliferative effect (28, 74–77). Native somatostatin is rapidly metabolized and has a short half-life (1–3 min) *in vivo*, which limits its clinical use, whereas synthetic somatostatin analogs like octreotide are much more stable (20). Somatostatin analogs have already achieved promising effects in the treatment of high-SSTR2-expression tumors, such as gastroenteropancreatic neuroendocrine tumors and GH-secreting pituitary adenomas (78, 79). The therapeutic efficacy of somatostatin analogs for meningiomas *in vitro* has been confirmed in various studies (27, 77, 80, 81). For example, Graillon et al. demonstrated that octreotide significantly decreased proliferation in 88% of fresh primary meningioma cells (82). Nonetheless, octreotide has been shown not to induce apoptosis of meningioma cells (82).

**FIGURE 2:**
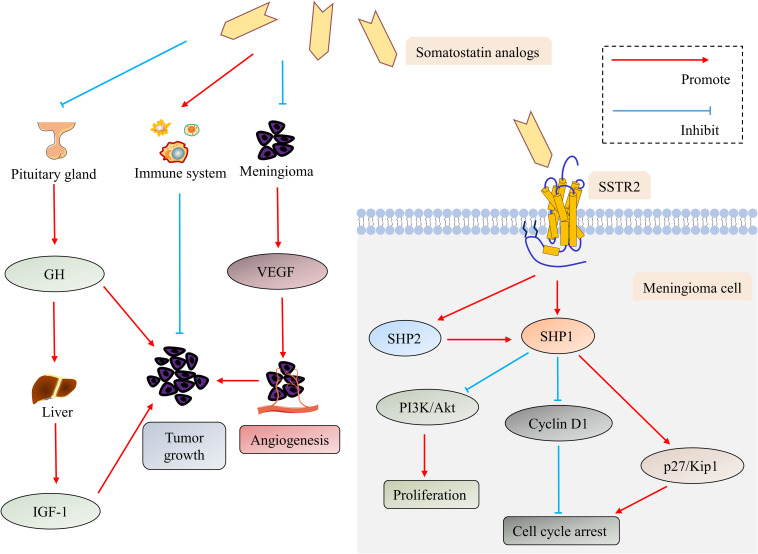
The direct and indirect antitumor mechanisms of the somatostatin analogs in meningioma. Somatostatin analogs exert their direct antitumor effects by binding to SSTR2, which leads to the activation of SHP1 and SHP2. SHP2 can further activate SHP1. SHP1 mediates antiproliferative action through inhibiting the PI3K/Akt pathway and induces cell cycle arrest through down-regulating cyclin D1 while up-regulating p27/Kip 1. Suppressing secretion of VEGF and GH/IGF-1 and activating the immune system are involved in indirect antitumor mechanisms of somatostatin analogs. Abbreviations: Akt, protein kinase B; GH, growth hormone; insulin-like growth factor-1 (IGF-1); PI3K, phosphatidylinositol 3-kinase; SHP1, SH2-containing phosphatase-1; SHP2, SH2-containing phosphatase-2; SSTR, somatostatin receptor; and VEGF, vascular endothelial growth factor.

The direct and indirect antitumor mechanisms (Figure 2) of the SSTR2 ligands–somatostatin analogs for the treatment of meningioma have been explored in several preclinical researches. Somatostatin or its analogs bind to SSTR2, leading to the activation of specific tyrosine phosphatases (SHP1 and SHP2) and the inhibition of the PI3K/Akt pathways, which mediate its direct antitumor effects through the induction of cyclin-dependent kinase inhibitors and cell cycle arrest (27, 80, 81, 83–87). The indirect antitumor mechanisms of somatostatin analogs incorporate (1) reduction of angiogenesis, particularly by inhibiting vascular endothelial growth factor (VEGF) secretion; (2) suppression of growth factors and hormone secretion that will drive tumor growth; and (3) stimulation of natural antitumor mechanisms (27, 84, 86–88). The synthesis of VEGF, one of the dominant proangiogenic factors, was decreased in meningioma cells by somatostatin analogs, indicating their antiangiogenic effects (27, 84, 86, 87). Somatostatin analogs can inhibit the release of GH from the pituitary gland, which causes the suppression of hepatic production of insulin-like growth factor-1 (IGF-1) (84, 86, 87). Both GH and IGF-1 have been proven to be tumor-promoting factors for meningioma (84, 86–88). Somatostatin and its analogs are also capable of activating the immune system, for SSTR2 are expressed in some immune cells (84, 86, 87, 89).

In some cases, considerable efficacy of somatostatin analogs could even be achieved in the treatment of unresectable or recurrent meningiomas (90–92). Rammo et al. reported a patient with progressive anaplastic meningioma treated with octreotide. Prior to octreotide therapy, repeated surgery and radiation therapy did not help stop the progression of the disease, but surprisingly, this patient remained in remission for over 3 years following octreotide treatment (90).

A few clinical studies have been carried out to evaluate the efficacy and safety of somatostatin analogs in the treatment for patients with meningioma (Table 3). A prospective pilot trial was carried out by Chamberlain et al. with a sustained-release somatostatin analog (Sandostatin LAR) treating 16 recurrent meningioma patients. The median overall survival (OS) was 7.5 months; 31% of patients achieved partial radiographic response, and 44% achieved 6 months progression-free survival (PFS); the toxicity of Sandostatin LAR was small (74). These results revealed that Sandostatin LAR might be a useful and tolerable alternative therapy option for recurrent meningiomas. Johnson et al. conducted a phase II study of subcutaneous octreotide treatment for recurrent meningioma patients. The results of this study were less satisfactory: even though octreotide was well tolerated and 2 of 11 patients experienced prolonged stability, it had not been able to produce objective tumor response (93). Complete resection of skull base meningiomas is always challenging; to this end, Schulz et al. treated patients harboring a progressive residual meningioma after surgery with a somatostatin analog. Disregarding the fact that no case of tumor disappearance was observed, the disease appeared to have stabilized in all cases (94). This study offered a perspective on additional therapy for post-surgery skull base meningiomas with somatostatin analogs. Regretfully, it was not a randomized controlled prospective clinical trial. For the treatment of recurrent high-grade meningioma, the efficacy of somatostatin analog might be limited, according to a phase II study showing that none of nine patients achieved radiographic partial response (82). In another trial, a somatostatin analog called pasireotide LAR (SOM230C) was prescribed monthly to patients with recurrent or progressive meningioma; unfortunately, it also failed to increase the proportion of patients with 6 months PFS significantly (95). Studies have manifested that the low levels of Raf kinase inhibitory protein or the mutations of aryl hydrocarbon receptor interacting protein were related to the unsatisfactory response to somatostatin analogs for the treatment of GH-secreting pituitary adenomas (96, 97), notwithstanding the fact that there is a paucity of similar studies in meningioma. Taken collectively, somatostatin analogs represent a safe but undefined therapeutic option in meningioma management. Notably, these clinical trials suffer from limited sample size and short duration, so more and larger trials are urgently warranted.

**TABLE 3:** Clinical studies using somatostatin analogs for meningioma treatment.

Subjects	No. of pts	Somatostatin analog	Dose	mTCs	Tumor response			Survival			TT due to AEs	References
					PR	SD	PD	PFS6	mPFS/TTP (months)	mOS (months)		
Recurrent M	16	Sandostatin LAR	30 mg/4 weeks	4.5	5	5	6	44%	5	7.5	0	(74)
Recurrent high-grade M	9	Sandostatin LAR	30–40 mg/4 weeks	3	0	3	6	44%	4.2	18.7	0	(82)
Recurrent or progressive M	11	Sandostatin LAR	500 μg 3 times/day	NA	0	8	3	30%	3.9	NA	0	(93)
Progressive residual M after surgery	8	Sandostatin LAR	30 mg/4 weeks	NA	0	8	0	100%	NA	NA	1	(94)
Recurrent or progressive M	34	Pasireotide LAR	60 mg/4 weeks	NA	0	24	8	32%	4.2	NA	0	(95)

*AEs, adverse events; M, meningioma; mOS, median overall survival; mPFS, median progression survival; mTCs, median treatment cycles; NA, not-available; PFS6, 6 months progression survival; PR, partial response; Pts, patients; PD, progressive disease; SD, stable disease; TT, treatment interruption; and TTP, time to progression.*

**FIGURE 3:**
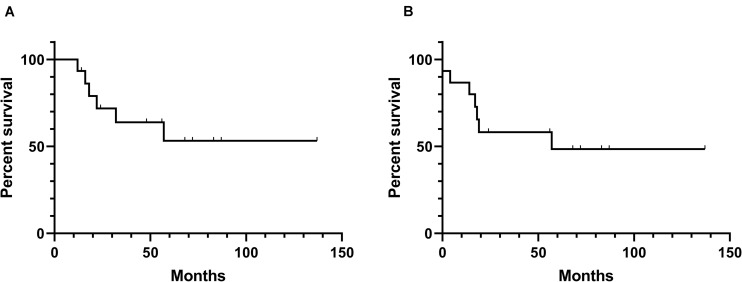
Kaplan–Meier curves of overall survival and progression-free survival of the reported effects (101) of SSTR-related radiation therapy. **(A)** Overall survival. **(B)** Progression-free survival.

SSTR2-directed peptide receptor radionuclide therapy (PRRT) has also exhibited their potential therapeutic use for patients with meningiomas. Beta-emitters 90-yttrium (^90^Y) and 177-lutetium (^177^Lu) are the most widely used radiometals in PRRT at present (98). Certain amounts of clinical studies (Table 4) have investigated the therapeutic effect of SSTR2-targeted PRRT in treating meningioma patients. In a clinical study, five meningioma patients, among which three had tumors that were very large with standard medical therapies that all failed, were treated with ^177^Lu-octreotate. Consequently, two of them had stable disease (SD) while three of them had progressive disease after PRRT treatment (99). A retrospective study also presented the activity of SSTR2-targeted PRRT using ^177^Lu-DOTATATE or ^90^Y-DOTATOC in patients with meningioma, with the results that 10 of 20 patients achieved SD for a median time of 17 months (100). Gerster-Gilliéron et al. have recommended ^90^Y-DOTATOC as a second- or third-line option for recurrent or progressive meningiomas, since median PFS (Figure 3) of patients receiving systemic ^90^Y-DOTATOC treatment was 57 months and the treatment was safe (101). Moreover, the results of a phase II prospective clinical trial, in which 67.6% of patients achieved SD and the mean survival of all enrolled patients was 8.6 years, lent further support to the use of SSTR2-directed PRRT in patients with progressive unresectable meningioma (102). Many more clinical studies have confirmed the efficacy and safety of SSTR2-targeted PRRT in the treatment of meningiomas (103–105). Indeed, the recent European Association of Neuro-Oncology guidelines on meningioma have declared PRRT a promising approach to treat refractory meningiomas across all WHO grades in the future (1). The selective accumulation of radiolabeled somatostatin analogs in meningioma cells enhances the efficacy while reducing the toxicity of PRRT. Nevertheless, because these traces are mainly excreted by the kidney, renal toxicity seems inevitable, which may limit the application of PRRT (98, 106, 107). Generally, in patients with recurrent or complex unresectable meningiomas, especially in those where standard treatments have failed, the use of SSTR2-targeted PRRT should be considered; for those who accept PRRT, we should pay close attention to their renal function, and renal protection should be provided.

**TABLE 4:** Clinical studies of PRRT treatment for meningioma.

Subjects	No. of pts	Intervention	Dose (GBq)	Cycles	Tumor response				mPFS (months)	Other main results/conclusions	References
					CR	PR	SD	PD			
Recurrent or progressive Ms	5	^177^Lu-octreotate	14.8–29.6	2–4	0	0	2	3	NA	177Lu-octreotate can have therapeutic effects in meningioma.	(99)
Progressive Ms	20	^177^Lu-DOTATATE ^90^Y-DOTATOC	13.7–27.6	1–4	0	0	10	10	5.4	PFS6 was 42%; treatment was well tolerated.	(100)
Recurrent or progressive Ms	15	^90^Y-DOTATOC	1.35–14.8	2–4	0	0	13	2	24	Toxicity was moderate.	(101)
Progressive unresectable Ms	34	^177^Lu-DOTATATE ^90^Y-DOTATOC	1.5–22.2	1–4	0	0	23	11	NA	The mean survival 8.6 years; treatment was well tolerated,	(102)
Recurrent Ms	29	^90^Y-DOTATOC	5–15	2–6	0	0	19	10	21	Median OS was 40 months; treatment was well tolerated.	(103)
Recurrent Ms	8	^111^In-Pentetreotide	4.8–29	2–4	0	2	5	1	NA	Treatment was well tolerated.	(104)
Advanced symptomatic Ms	10	EBRT + ^177^Lu-DOTATATE or DOTATOC	7.0–7.9	1	1	1	8	0	13.4	The combination treatment was safe and effective.	(105)

*CR = complete response; DOTATOC = DOTA-(Tyr3)-octreotide; DOTATATE = DOTA-D-Phe1-Tyr3-octreotate;EBRT = External beam radiotherapy; 111In = 111-Indium; 177Lu = 177-Lutetium; mPFS = median progression survival; Ms = meningiomas; NA = not-available; OS = overall survival; PD = progressive disease; PFS6 = 6 months progression survival; PR = partial response; Pts = patients; SD = stable disease; 90Y = 90-Yttrium.*

Taken together, SSTR2-related/targeted treatments are promising approaches for the treatment of unresectable or refractory meningiomas. Somatostatin analogs can only inhibit the proliferation but fail to induce the apoptosis of meningioma cells; meanwhile, somatostatin analog treatment for meningioma exhibits efficacy *in vitro* and some special cases, but clinical studies have not achieved satisfactory results. Consequently, the effectiveness of somatostatin analog treatment for meningioma is actually controversial currently; further studies are required to identify and select the patients in whom treatment with somatostatin analogs is potentially effective. Importantly, SSTR2-targeted PRRT has shown an effect on the treatment of meningiomas in some clinical studies.

## SSTR2 in Prognostic Prediction of Meningioma

It is of clinical importance to predict the prognosis of meningioma patients, since it can provide a valuable reference for the proper management of patients, such as making treatment and follow-up strategies. Previous studies have manifested several potential prognostic indicators for meningioma, including the WHO tumor grade, the extent of resection, expression of progesterone and estrogen receptors, mitotic index, and bone involvement (72, 108–111). However, additional prognostic factors are still sorely needed to better predict the outcomes of meningioma patients.

Barresi et al. have attempted to draw the association between SSTR2 and tumor grade by analyzing SSTR2 immunohistochemical expression in 35 different-grade meningiomas; their results have shown that SSTR2 was frequently expressed in high-grade meningiomas and related to higher microvessel density (30). Explicitly, 57% grade I, 75% grade II, and 66% grade III meningiomas were characterized by a high expression of SSTR2 (30). Somatostatin or its analogs might be effective in the therapy of meningiomas by reducing their blood supply based on this study (112). Nevertheless, Durand et al. have found that SSTR2 levels were not grade related but histotype related, with significantly higher expression levels in the meningothelial subtype than in the fibroblastic subtype (29). This finding may support the use of somatostatin or its analogs to treat this subtype. Silva et al. have argued that SSTR2 levels might correlate to the risk of recurrence because the high expression of SSTR2 was observed in partially resected meningiomas with tumor regrowth (25). Additionally, Seystahl et al. have also observed that the expression level of SSTR2 was not correlated with the WHO grade of meningiomas; yet the expression level of SSTR could be a predictive biomarker for the outcome of meningioma patients treated with PRRT; a higher expression of SSTR2 was revealed to be associated with better PFS after PRRT treatment (100). These researches indicate that whether SSTR2 levels are grade related in meningiomas remains controversial; meanwhile, SSTR2 could still offer some implications for prognosis prediction in spite of this controversy.

## Conclusion and Prospects

Meningiomas are the most frequent intracranial tumors. SSTR2 expressed in almost all meningiomas, which provides novel ideas and approaches in the diagnosis, treatment, and prognostic prediction for meningiomas. Certain progress regarding the clinical significance of SSTR2 in meningioma has been made in the past few decades. SSTR2-related imaging tools with radiolabeled somatostatin agonists, including PET, PET/CT, PET MRI, SPECT SRS, and SPECT/CT SRS, have significant value in (preclinical) diagnosis, differential diagnosis, and disease evaluation. Despite accumulating evidence that SSTR2-related/targeted treatments (e.g., somatostatin analogs and SSTR2-targeted PRRT) are promising and safe therapeutic options for unresectable or refractory meningiomas, several controversial areas remain. More and larger multicenter long-term follow-up and randomized prospective trials are urgently needed, especially in uncovering the precise underlying signaling pathways of SSTR2 ligands–somatostatin analogs’ antitumor effects as well as identifying and selecting candidate patients who may benefit from these treatments.

## Author Contributions

YZ, WW, and AS conceptualized the research project. WW, YZ, YW, and LL drafted the manuscript. YW and JL drew the figures. PZ and AS reviewed and modified the manuscript. AS, YD, and PZ supervised the research and led the discussion. All authors approved the final version of the manuscript.

## Conflict of Interest

The authors declare that the research was conducted in the absence of any commercial or financial relationships that could be construed as a potential conflict of interest.

## Abbreviations

^111^ In: 111-indium
^177^Lu: 177-lutetium
^90^Y: 90-yttrium
CNS: central nervous system
CT: computed tomography
DOTATATE: DOTA-D-Phe1-Tyr3-octreotate
DOTATOC: DOTA-(Tyr3)-octreotide
IGF-1: insulin-like growth factor-1
MRI: magnetic resonance imaging
PET: positron emission tomography
PFS: progression-free survival
PRRT: peptide receptor radionuclide therapy
SD: stable disease
SHP1: SH2-containing phosphatase-1
SHP2: SH2-containing phosphatase-2
SPECT: single-photon emission computed tomography
SRS: somatostatin receptor scintigraphy
SSTR: somatostatin receptor
VEGF: vascular endothelial growth factor
WHO: World Health Organization.
